# Supplementary material for: Are carbon emissions trading and green financial instruments synergistic? -Comprehensive quantitative research based on content analysis
Source: PLoS One. 2024 Mar 7;19(3):e0298601. doi: 10.1371/journal.pone.0298601 (PMC10919601; doi:10.1371/journal.pone.0298601)
Supplement: S1 Data — (ZIP) [file pone.0298601.s001.zip › Data/地级市Z_F工作报告.zip!/╜¡╦╒/╦╒╓▌╩╨/└┤╫╘╣1⁄2╓┌║┼í╛┬φ┐╦╩2╛▌═°í┐_2012.docx]

各位代表：

现在，我代表苏州市人民政府，向大会报告工作，请予审议，并请市政协委员和其他列席人员提出意见。

一、本届政府任期以来的工作回顾

2008年以来，在中共苏州市委的带领下，市政府和全市人民主动顺应宏观形势变化，积极应对国际金融危机等严峻挑战，努力克服前进中遇到的种种困难，巩固提升小康建设成果，奋力开启率先基本实现现代化新征程，经济社会发展取得了一系列成就。

一是致力于率先发展、转型升级，经济总量连续攀升，运行质量持续提高。全面推进产业发展，切实加强自主创新，经济实现较快增长。2008年～2011年，地区生产总值年均增长12.5%，地方一般预算收入年均增长19.4%，总量分别跨入万亿元级和千亿元级行列；工业总产值年均增长14.8%，2011年超过3.3万亿元，保持全国第二大工业城市地位；全社会固定资产投资年均增长17.4%，社会消费品零售总额年均增长19.9%，2011年分别达到4502亿元和2830亿元，位居全国前列。产业结构得到优化，第一、第二、第三产业增加值比例由2∶62.1∶35.9调整为1.7∶55.6∶42.7。服务业快速发展，增加值达到4582亿元，占地区生产总值的比重比2007年提高6.8个百分点。金融、物流、会展等生产性服务业规模不断扩大，商贸、旅游等生活性服务业层次进一步提高。新兴产业成为新的增长点，产值占到规模以上工业的38.7%。高新技术产业产值年均增长14.3%，2011年达到1.1万亿元。传统产业改造提升，竞争实力逐步增强。做大做强地标型企业，百强工业企业销售收入占规模以上工业的比重达到41.8%。现代农业做精做优，建成万亩以上农业示范园区23个，高效农业比重超过60%，农业现代化水平位居全省首位。我市成为国家创新型城市建设试点市，科技进步统计监测综合评价连续四年保持全省第一。独墅湖科教创新区加快建设，国家知识产权局专利审查协作江苏中心、浙江大学苏州工业技术研究院顺利落户，清华大学苏州汽车研究院（吴江）启动建设。中科院纳米技术与纳米仿生研究所一期、生物医学工程技术研究所一期等重大创新载体相继建成，63%的本土大中型企业设立研发机构，公共技术服务平台不断健全。全社会研究与试验发展经费支出占地区生产总值的比重达到2.45%，比2007年提高0.73个百分点。全市人才总量超过160万人，高层次人才数量达到9.7万人，分别是2007年的1.7倍和1.9倍；设立国家“千人计划”创投中心，81人入选国家“千人计划”，居全国地级市首位。质量强市、商标品牌、技术标准、知识产权等工作全面加强，有力地促进了经济转型发展。

二是致力于深化改革、扩大开放，体制机制不断完善，发展活力明显增强。推进制度创新，提高开放水平，经济社会发展更具生机活力。国有经济战略性调整积极实施，监管体系逐步健全，有效实现保值增值。行政审批制度改革取得进展，市级机关行政审批总事项削减71%。社会建设和管理切实加强，城市管理体制、社区管理体制改革力度加大。医药卫生体制改革扎实开展，政府办基层医疗卫生机构全部实施国家基本药物制度。金融改革创新成果显著，苏州银行和东吴人寿保险公司先后成立，东吴证券公司成功上市，苏州产权交易中心投入运营，备案创业投资企业达到88家，新增上市企业42家，累计达到70家。民营经济迅速发展，私营企业和个体工商户由2007年的38.6万户增加到59.1万户，净增注册资本4585亿元，总额达到7506亿元。开放型经济保持领先优势，进出口总额年均增长9.2%，实际利用外资继续位居全国前列，中方境外投资额实现全省“八连冠”，国家级境外经贸合作区埃塞俄比亚东方工业园建设稳步推进。苏州工业园区等开发区建设成效明显，5个省级开发区升格为国家级开发区。全市新结国际友好城市5对，累计达到43对。外事、对台、侨务等工作再创新业绩，服务经济社会发展的能力持续提升。共建园区建设进展顺利，南北挂钩、对口援建帮扶工作积极开展。

三是致力于统筹规划、持续发展，城乡一体进程加快，生态环境逐步优化。加大基础设施建设力度，深化城乡一体化发展综合配套改革，严格保护生态环境，协调发展的能力得到增强。城市规划编制水平不断提高，重点地区、重点项目规划有效实施。沪宁城际铁路、京沪高速铁路苏州段建成通车，苏州火车站北站房及配套设施投入使用。每百平方公里高速公路里程增至6.3公里，达到发达国家水平。苏州港年吞吐量跃至全国港口第五位、连续多年稳居内河港第一位。环金鸡湖金融商贸区已具规模，平江、沧浪、金阊新城基本形成城市形态，滨湖新城、高铁新城规划建设全面启动。城乡电网建设实现新的跨越，供水、供气得到有效保障，社会信息化水平明显提升。民防设施继续完善，防震减灾避险能力进一步增强。城市管理长效机制全面落实，打造全国最干净城市创建活动收到实效。城乡一体化大力推进，我市成为全国农村改革试验区和全省城乡一体化发展综合配套改革试点市。公共财政对“三农”的投入逐年增加，城乡一体的政策制度框架基本建立。城乡公共服务均等化和社会保障并轨步伐加快，社区服务中心实现全覆盖。社区股份合作、土地股份合作和农民专业合作组织快速发展，农村新型集体经济得到壮大。城乡资源配置和空间布局不断优化，88%的农村工业企业进入工业园，80%的承包耕地实现规模经营，43%的农户迁入集中居住点，110多万农民实现居住地向城镇转移。新一轮村庄环境整治积极开展，农村环境逐步改善。更加注重环境保护，着力加强重点流域、重点区域、重点行业环境整治，水环境质量和空气质量有所提高，声环境功能区噪声稳定达标，环境质量综合指数稳步上升。发展循环经济，推行清洁生产，节约利用资源，省下达的节能减排任务超额完成。广泛开展城乡绿化，石湖景区滨湖区域等一批重大绿化景观项目建成使用。建立生态补偿机制，严格保护生态资源，生态建设取得重要进展。国家环境保护模范城市通过复核，创建国家生态市通过考核验收。

四是致力于协调提升、全面进步，社会事业繁荣发展，公共服务继续加强。坚持教育优先，教育现代化迈出新步伐。注重素质教育，强化内涵建设，促进学生全面发展。学前教育的公益性、普惠性得到增强，公办义务教育学校全部建成教育现代化学校，公办高中实现三星级以上全覆盖，高等教育、职业教育、继续教育办学水平普遍提高。文化苏州建设再结硕果，一批重大文化项目建成使用，公益性文化设施基本实现市、县级市（区）、镇、村全覆盖。群众文化活动蓬勃开展，文艺创作精品迭出，我市文艺作品和表演艺术家屡获全国大奖。切实保护文化遗产和地方历史文化，注重在保护中传承、在传承中发扬。虎丘地区综合改造、桃花坞历史文化片区综合整治保护利用工程有力推进，木渎春秋古城遗址考古发掘顺利实施。第二轮《苏州市志》完成总纂。6家档案馆晋升为国家一级档案馆。文化产业体系逐步健全，建成国家级、省级文化产业示范基地11个。文化旅游加快发展，我市5A级景区数量居全省第一，工农业旅游示范点数量居全国地级市之首。城乡医疗卫生服务体系基本形成，医疗综合服务能力持续增强，健康城市建设深入推进，全民健身服务工程积极实施，市民平均期望寿命达到81.56岁。苏州体育健儿在北京奥运会等国际大赛上夺得16次世界冠军。人口和计划生育管理服务再上新水平。妇女儿童工作取得新成绩。民族团结进步的局面进一步巩固，宗教事务管理规范有序。国防动员和国防后备力量建设不断强化，双拥共建工作取得成效，优抚安置政策全面落实，新型军政军民关系更加紧密，我市创建全国双拥模范城实现“五连冠”。

五是致力于以人为本、民生优先，人民生活有效改善，群众得到更多实惠。大力推动就业惠民、创业富民、社保安民，持之以恒抓好各类实事项目，保障和改善民生取得成效。城镇居民人均可支配收入年均增长11.8%，农民人均纯收入年均增长13.2%。多形式促进充分就业，新增城乡就业61.2万人，2011年城镇登记失业率控制在2.72%，好于全国全省水平。社会保障体系渐趋完善，城镇职工养老、医疗、失业、工伤、生育五大社会保险参保覆盖率，以及城乡居民基本养老保险覆盖率、被征地农民纳入社会保障覆盖率、新型农村合作医疗覆盖率均保持在98%以上，养老保险、医疗保险城乡并轨进程加快。社会福利制度由补缺型向适度普惠型转变，城乡最低生活保障标准自然增长机制启动实施，在全国率先实现城乡低保标准并轨。社会养老服务水平继续提升，残疾人社会保障和服务体系初步建立。惠民殡葬政策全面实行。保障性住房建设、城区居民家庭“改厕”、南环新村解危改造、危旧房和城中村（无地队）改造、老住宅小区和街巷综合整治等工程积极推进，住房公积金受益面持续扩大。对市区义务教育阶段学生、残疾人、70周岁以上老年人提供免费乘坐公交和轨道交通待遇。为老年人开展了两轮免费健康体检。建立健全食品、药品监管网络，构建肉类蔬菜流通追溯体系，严厉查处危害群众健康的违法行为。建成重要民生商品价格采集发布系统，设立农产品平价直销网点，适时发放临时物价补贴，保供稳价措施有效落实。

六是致力于创新管理、促进和谐，精神文明、民主法治和政府自身建设取得新的进展。开展“讲文明、树新风”主题教育实践活动，发扬苏州城市精神，加强未成年人思想道德建设，强化文明城市长效管理，我市荣膺并蝉联全国文明城市称号，张家港市成为唯一的全国文明城市“三连冠”县级市。科学知识广泛普及，哲学社会科学研究再获新成果。深入推进法治苏州建设，加大法治县（市、区）创建力度，开展普法教育，健全法治惠民机制，基层民主法治水平逐步提高，全社会法治意识普遍增强。平安苏州建设切实加强，社会治安秩序持续好转，人民群众安全感不断提升，我市荣获全国社会治安综合治理优秀市“五连冠”，并再次被授予“长安杯”。建立社会矛盾纠纷排查化解体系，信访工作走向规范化、法制化，一批历史遗留问题得到妥善处理。进一步落实安全生产责任，挂牌整改重大事故隐患2554项，各类事故控制指标连年下降。市、区应急管理工作机构和网络全面建立，应急综合演练实现常规化、制度化。认真做好政府立法工作，本届政府共提请市人大常委会审议通过地方性法规13件，颁布政府规章24件。坚持依法行政，强化行政监督，推进科学民主决策。新一轮政府机构改革顺利完成，经济发达镇行政管理体制改革试点稳步开展。作风效能建设扎实深入，行政效率和服务质量进一步提高。反腐倡廉取得新成效，初步建成具有苏州特点的惩治和预防腐败体系框架。推进行政权力公开透明运行，政务公开的内容与范围不断扩大。加强审计监督，促进了公共资金规范、安全、高效使用。市政府自觉接受市人民代表大会及其常务委员会的法律监督、工作监督和市政协的民主监督，坚持重大事项向市人大常委会报告、向市政协通报，注重发挥各民主党派、工商联、无党派人士和人民团体的参政议政作用。组织实施人大议案6件，办复代表建议862件、政协提案1883件。

今年以来，我们围绕市十四届人大五次会议确定的年度目标，突出加快转型、稳定外贸、兴盛文化、广惠民生等重点，按照稳中求进、又好又快的工作导向，振奋精神、迎难而上，求真务实、奋力拼搏，推动了经济社会健康发展。虽然经济下行压力加大，但经过全市各个方面的共同努力，总体呈现低开稳走、逐步向好的态势，产业结构得到优化，现代服务业和新兴产业加快发展。社会事业全面进步，和谐融洽局面进一步巩固。民生质量持续改善，城乡居民收入增长较快。城市轨道交通1号线投入试运营，中环快速路开工建设，实事项目进展顺利。改革发展稳定的各项任务都在抓紧落实，政府工作达到时序进度要求。

各位代表，苏州的发展历程令人难忘，发展成就令人振奋。这是全市人民在中共苏州市委的正确领导下，同心同德、团结奋斗的结果，是市人大、市政协加强监督、鼎力支持的结果。在此，我代表市政府向全市人民，向人大代表、政协委员，向离退休老同志，向各民主党派、工商联和无党派人士，向各人民团体，向驻苏解放军和武警官兵，向国家和省驻苏单位，向所有参与、支持和关心苏州现代化建设的海内外朋友，表示衷心的感谢和崇高的敬意！

同时，我们也看到，经济社会发展中还存在不少问题，政府工作与人民期待相比仍有差距，主要是：经济发展方式尚未实现根本转变，经济结构不尽合理，产业层次有待提高；人才结构性矛盾较为突出，创新创业人才仍然缺乏，自主创新能力亟需提升；中心城区功能偏弱，城市综合承载能力还要增强；人口、资源、环境压力较大，土地、能源等瓶颈制约加剧；城乡居民富裕程度、社会事业发展水平与人民群众日益增长的物质文化需求存在差距；新型社会矛盾增多，社会建设与管理任务加重；政府职能需要加快转变，公共服务还需进一步加强。对此，我们将予以高度重视，采取有效措施，认真加以解决。

二、新一届政府工作的总体思路和主要目标

综观国内外形势，我们仍处于大有可为的重要战略机遇期，既面对诸多风险挑战，更面临难得的历史机遇。可以预见，今后一个时期，世界经济形势严峻复杂，不确定、不稳定因素仍然较多，经济复苏的动力明显不足；国内区域发展格局正在发生重大而深刻的变化，以加快经济转型升级为重点的地区之间、城市之间的竞争日趋激烈。而总的来看，经济社会保持又好又快发展的基本条件和长期趋势不会改变。经济全球化深入发展，科技革命突飞猛进，世界范围内经济重组和产业转移步伐加快，有助于我们在更广领域内、更高层次上参与国际分工与合作。我国工业化、城镇化、信息化快速推进，消费结构和产业结构升级蕴藏着巨大的需求潜力。长三角区域一体化进程加速，苏南现代化示范区建设已经提上议事日程，必将推动区域内城市优势互补、合作共赢。进入新世纪以来，我市大力推进“两个率先”，成功绘就了全面小康的现实模样，率先基本实现现代化取得可喜进展，这为今后发展创造了良好条件、奠定了坚实基础。

根据中共苏州市第十一次党代会精神，建议新一届政府工作的总体思路是：高举中国特色社会主义伟大旗帜，以邓小平理论和“三个代表”重要思想为指导，以科学发展观统领全局，紧紧围绕“三区三城”和“建设宜居新苏州、打造创业新天堂、共筑幸福新家园”的总定位总要求，深入实施创新引领、开放提升、城乡一体、人才强市、民生优先、可持续发展战略，突出转型升级、富民惠民、崇文重教、统筹发展等重点，在率先基本实现现代化、全面完成“十二五”规划任务的基础上，朝着更高水平的现代化目标阔步迈进。

建议新一届政府任期内经济社会发展的预期目标是：

主要经济指标全国领先。地区生产总值年均增长12%左右，人均达到2.4万美元左右。地方一般预算收入与地区生产总值基本同步增长。投资、消费、出口三大需求实现结构优化、协调增长，投资规模继续扩大，消费对经济增长的贡献率明显提高，对外贸易保持量增质优态势。

产业竞争能力全国领先。形成以服务经济为主导的产业结构，服务业增加值占地区生产总值的比重达到50%左右；新兴产业产值、高新技术产业产值占规模以上工业的比重分别提高到50%和43%以上；现代农业再上新水平，高效农业比重达到66%，生态、生产、生活、生物功能得到充分发挥。全社会研究与试验发展经费支出占地区生产总值的比重超过3%，科技进步对经济增长的贡献率超过65%，人才总量超过220万人，建成国家创新型城市。

人民生活水平全国领先。城镇居民人均可支配收入年均增长12%，农民人均纯收入实现更快增长，城乡收入之比缩小到1.9∶1。就业形势保持稳定，城镇登记失业率、社会登记失业率均控制在4%以内。社会事业全面进步，文化生活丰富多彩，社会保障达到更高水准。

城乡一体发展全国领先。城乡发展规划、产业布局、资源配置、基础设施、公共服务、就业社保和社会管理一体化格局更为完善。城乡建设统筹推进，各项事业共同发展，村镇面貌明显改观，广大农民普遍受益，农村现代化水平大幅度提升。

生态环境改善全国领先。水、气、声等环境和耕地质量全面优化，环境质量综合指数继续攀升。陆地森林覆盖率达到27.5%，市区建成区绿化覆盖率、绿地率分别达到44%和38.5%。单位地区生产总值能源消耗下降、主要污染物排放总量削减和排放强度下降均完成省下达的任务。

和谐社会建设全国领先。社会主义核心价值观深入人心，市民文明素质和社会文明程度进一步提高。民主法制更加健全，依法行政全面加强，社会管理不断完善，诚信体系基本建立，公平正义得到维护。社会安定有序，人民群众安全感显著增强。

新形势新任务对政府工作提出了更高要求。建议新一届政府认真总结成功经验，科学谋划未来发展，在今后工作中把握好以下几点：

一是坚持改革开放，促进率先发展。坚定不移地深化改革，革故鼎新，破除障碍，建立健全有利于科学发展的体制机制。拓展对外对内开放的广度和深度，形成以国际化企业为主体、国际化城市为基础、国际化人才为支撑的新格局，增强苏州的国际竞争力和影响力。突出科学发展主题，紧扣转变经济发展方式主线，当好“两个率先”的排头兵和先行军。

二是坚持结构优化，促进转型发展。切实做到先进制造业与现代服务业“双轮驱动”，新兴产业增量扩张与传统产业存量提升“两产并举”，工业化与信息化“两化融合”，推进产业高端化、技术高新化。自觉地将自主创新贯穿于现代化建设的各个领域、各个方面，推动经济社会又好又快发展。深刻认识人才是第一资源，营造引得进、留得住、用得好的人才环境，形成人才辈出、人尽其才、才尽其用的生动局面。

三是坚持成果共享，促进为民发展。牢牢把握以人为本的核心要义，始终情系人民，时刻想着人民，紧紧依靠人民，一切为了人民，使全市人民普遍共享改革发展成果。以扩大就业、促进创业为着力点，推动城乡居民持续增收，形成与经济发展相协调的收入增长机制。加强社会保险、帮困救助、慈善福利之间的衔接，构建更为完善的社会保障体系。积极创造条件，让每个人都能够各展所长、各尽所能追求自己的幸福生活，同时也为增进全社会的福祉贡献力量。

四是坚持统筹兼顾，促进协调发展。妥善处理经济与社会、城市与农村、人与自然等重大关系，更加注重全面均衡发展。加大社会事业投入力度，努力实现经济发展与社会进步相统一。加强城乡统筹，努力实现城市发展与农村发展相融合。始终不渝地走生产发展、生活富裕、生态良好的可持续发展之路，努力实现物质文明建设与生态文明建设相同步。

五是坚持着眼未来，促进长远发展。立足更高要求、更高标准，制定、完善和落实各项发展规划、建设规划。遵循公众意愿，顺应市场需求，筹划、建设并储备一批符合国家产业政策、具有前瞻性、事关全局和长远的重大项目；严格保护并高效利用土地等各类资源，为今后发展增添后劲、积蓄能量、留出空间。

六是坚持凝心聚力，促进创新发展。弘扬崇文、融和、创新、致远的城市精神，丰富和发展“张家港精神”、“昆山之路”和“园区经验”，不断创造苏州特有的精神财富，汇聚全市人民奋发向上的精神力量，激发全社会的创新激情、创业豪情、创优热情。与时俱进，大胆实践，以攻坚克难的勇气、争先进位的志气、敢于探索的锐气，开辟发展新境界、开创工作新局面。

三、新一届政府的重点工作

建议新一届政府着重做好以下九个方面的工作：

（一）加快优化经济结构，构建高水平现代产业体系

跨越发展现代服务业，壮大提升先进制造业，精心培育优质高效农业。按照生产服务业集聚化、生活服务业便利化、基础服务业网络化、公共服务业均等化的要求，推动服务业发展提速、比重提高、质量提升。重点发展金融、物流、商务、会展、研发设计、软件与信息服务等生产性服务业，引进和集聚国内外大企业大集团的区域总部、功能总部。促进旅游、商贸等生活性服务业提档升级，建设商贸大市、旅游强市。加快推进保税物流园区、软件和服务外包产业园、创意动漫产业园等重要载体建设，扩容提升城市商圈，做大做强环金鸡湖金融商贸区、苏州高新区狮山商贸区、昆山花桥国际商务城、苏州太湖国家旅游度假区、江苏长江口旅游度假区等现代服务业集聚区。开展城市综合服务标准化试点，提高公共服务和专业服务水平。着力发展新能源、新材料、智能电网和物联网、高端装备制造等新兴产业和高新技术产业，尤其注重发挥纳米技术、新型平板显示等重大项目的引领带动作用，力争在总量扩张、产业链完善、核心技术研发应用、龙头企业成长壮大等方面取得更大突破，形成全国领先优势和国际竞争优势。实施“万企升级”行动计划，支持企业技术改造，提高电子信息、轻工、冶金等主导产业的发展水平，振兴丝绸、工艺美术等传统产业。推动产业梯度转移，为结构调整腾出空间。打造行业领先的特色产业基地、产业集群和拥有自主知识产权的地标型企业。鼓励企业加强品牌建设，争创更多驰名商标和名牌产品。坚持农场化、合作化、园区化发展方向，构建科学的产业布局、优美的生态环境、现代的科技支撑、完备的基础设施、安全的产品质量、健全的市场营销、高效的社会服务、完善的支持保护等八大体系，走出一条具有苏州特色的农业现代化道路。

（二）全力推动科技进步，积极争创国家创新型城市

坚持创新引领，增强科技、人才对经济社会发展的支撑能力和带动作用。集聚创新资源，完善以企业为主体、市场为导向、产学研紧密结合的自主创新体系。强化基层科技工作，依托重大科技项目和重点工程，注重资源与资本、科技与产业、产品与市场的结合，促进更多创新成果加速转化为现实生产力。构建开放共享的技术研发平台、检验检测平台、科技信息平台和技术转移平台，支持企业设立工程技术研究中心和技术中心。在创新科技体制机制上先行先试，积极参与和推动苏南自主创新示范区建设。加大政府对研发活动的支持力度，引导全社会增加科技投入，形成多元化的投入机制。推进科技与金融结合，扩大科技银行试点，鼓励发展科技小额贷款公司，做大科技金融规模。加强国际科技合作，吸引跨国公司来苏设立研发机构，引入先进技术、先进装备和先进管理方法，开展消化吸收基础上的再创新。抓好质量强市工作，鼓励企业参与重要技术标准研制，争创产业技术标准化示范区。优化知识产权法治环境，增强知识产权创造、运用、保护和管理能力，万人发明专利拥有量达到15件。落实国家“千人计划”、省高层次创新创业人才引进计划和“姑苏人才计划”，实施“1010工程”，继续办好国际精英创业周和“赢在苏州”等系列活动，深入开展“科技镇长团”派驻工作，推进国家海外高层次人才创新创业基地、国家“千人计划”创投中心和东沙湖股权投资中心建设。完善人才工作目标责任考核机制，放大人才工作优质品牌效应，引进培养高层次科技人才和现代服务业人才、高素质经营管理人才、高技能实用人才、高水平创新创业团队，以人才高地构筑创新高地、产业高地、竞争高地。

（三）纵深推进改革开放，不断激活发展的内生动力

通过深化体制改革破解发展难题，依靠提升开放水平拓展发展空间。分类实施事业单位改革，健全管理体制和人事、收入分配等制度。坚持公共医疗卫生的公益性质，继续推进医药卫生体制改革。鼓励符合要求的非政府办基层医疗卫生机构实施基本药物制度。开展资源性产品价格和环保收费改革，推行排污权许可证管理，建立排污权交易平台。发展要素市场，构建多层次市场体系，创造有效配置资源、促进转型升级的条件。加大金融改革创新力度，鼓励设立地方法人金融机构、股权投资基金和创业投资企业。力争我市成为全国服务业综合改革试点市和营业税改征增值税试点市。完善国有资产管理体制，优化国有经济战略布局，加快国有企业股份制改造步伐。拓宽民间资本投入领域，引导和支持民营经济做大做强，鼓励民营企业建立和完善现代企业制度，扶持小微企业成长。培育发展和规范管理社会组织，开展政府向社会组织转移职能的试点。在更高层次上推进对外对内开放，增创开放型经济新优势。以建设全国加工贸易转型升级试点市为契机，推动加工贸易向高端环节转型、向高附加值领域延伸。着眼于增强市场竞争力，努力形成集科技创新、品牌打造、基地建设、市场开拓于一体的一般贸易发展格局。以服务外包为重点，壮大服务贸易规模，发展电子商务。稳步扩大进口贸易，注重发展国际转口贸易、保税物流贸易和进口分销业务。更加重视招商选资与引技引智相结合，重点引进技术含量高、成长性好、安全无污染的创新型项目，在利用外资规模和质量上保持领先地位。促进外资企业与本土企业融合发展，实现研发本土化、生产本土化、人才本土化。注重“引进来”和“走出去”双向互动，大力培育本土跨国企业，积极开展服务业对外投资、境外资源开发和境外加工贸易，力争把埃塞俄比亚东方工业园建成境外经贸合作的样板区。推动开发区转型发展，实现体制优化、人才集聚、能级提升。提高中新合作水平，更好地发挥苏州工业园区的辐射带动作用。支持苏州高新区建设国家创新型科技园区。加快建设苏州工业园区——相城区合作经济开发区、海峡两岸（昆山）商贸合作区和中德（太仓）中小企业合作示范区。整合强化海关特殊监管区功能，探索设立具有自由贸易功能的经济合作园区。充分发挥外事、对台、侨务工作的重要作用，促进多层次、全方位、宽领域的对外交流与合作。争取与一批规模匹配、地位相当、优势互补的世界名城缔结为国际友城。推进苏宿、苏通、苏盐等共建园区建设。做好援藏、援疆等工作。

（四）着力加强建设管理，彰显精致秀美的城市特色

优化城市布局，增强承载能力，努力把苏州整体打造成为长三角重要的核心区域城市群。完善城镇体系规划，开展新一轮苏州市城市总体规划修编工作，注重城市规划与产业规划相互衔接，科学编制各类分区规划、详细规划、专项规划和城市设计。按照“一核四城”定位，做精做美做强古城核心，打造东部综合商务城，建设西部生态科技城、南部滨湖新城和北部高铁新城。完成苏州火车站地区综合改造任务，推动沪通铁路、沿江城际铁路和通苏嘉城际铁路建设。加密高等级公路，建成太仓疏港高速公路，基本完成张家港疏港高速公路和常嘉高速公路苏州段等工程。建成城市轨道交通2号线及延伸线、4号线，开工建设3号线，形成骨干线网，推动上海轨道交通11号线昆山延伸段建成运营，实现轨道交通与铁路、城市其他公交出行方式换乘的无缝对接。建成中环快速路和内环快速路放射线，构筑城市快速道路体系。推进以太仓港区为重点的苏州港整体发展，建设太仓港区集装箱码头四期工程。确保苏南运河、杨林塘、申张线航道升级整治和张家港复线船闸、通洲沙西水道综合整治等工程顺利竣工。完善防洪、供电、供水、供气等设施，健全防震减灾、应急避险和人民防空防护体系，实施气象监测和气象公共服务数字化工程。建设“城市光网”和“无线城市”，促进“三网融合”，打造“智慧苏州”，每千人国际互联网用户数达到1400户。建设国家历史文化名城保护示范区，完成虎丘地区综合改造、桃花坞历史文化片区综合整治保护利用、黎里等古镇保护开发工程，抓紧做好古城墙和古建老宅保护修缮工作，启动天赐庄历史文化片区保护更新项目，推进环古城河、大运河苏州城区段、胥江“两河一江”环境综合整治以及古城区河道水质提升等工程。完善城市管理体制机制，形成整体合力，切实加强道路交通、市政设施、公共绿地、建筑工地等管理，打造全国最干净、最整洁城市，塑造苏州良好形象。

（五）更加注重城乡统筹，加快形成一体化发展格局

深化城乡一体化发展综合配套改革，优化资源配置，打破二元结构。坚持规划引领，做到镇村布局、村庄建设、农业发展、乡村旅游、水网水系等规划紧密衔接，促进城镇建设、土地利用、产业发展、生态建设有机融合。继续推动农村工业企业向园区集中、农业用地向规模经营集中、农民居住向新型社区集中，规划建设好保留村庄。实行最严格的耕地保护制度，开展土地复垦整治，用好城乡建设用地增减挂钩政策。发展农村新型合作经济组织，健全农民增收长效机制，增加财产性收入，拓展经营性收入，提高工资性收入，巩固政策性收入。加强集体资产和集体经济管理，完善收益分配机制。加大强农惠农力度，确保财政投入总量、增量、比例持续提高。深化户籍制度改革，放宽县城和小城镇落户条件，鼓励引导农户将集体资产所有权、土地承包经营权、宅基地及住房置换成股份合作社股权、城镇保障和城镇住房，加快农民市民化、股民化、职业化步伐。完善城乡一体的就业社保、医疗卫生、教育文体、养老事业等服务体系，实现城乡公共服务均等化和社会保障一体化。提升农村道路、给水排水、绿化环卫、电力通讯等基础设施建设水平，做到城乡基础设施共建共享共用。创新农村金融服务，扩大农业贷款规模，拓宽政策性农业保险领域，增加农业担保种类，建立城乡一体化投资基金。高水准完成村庄环境整治任务，切实保护古镇古村落，显著改善农村面貌，既保持江南水乡的特色风情，又呈现先进和谐的现代文明。

（六）大力倡导崇文重教，推动文化教育实现新跨越

加快建设文化强市，优先发展教育事业。全面贯彻社会主义核心价值体系，巩固共同思想基础。广泛开展精神文明创建活动，力争率先建成全国文明城市群。健全未成年人思想道德教育体系。促进社会志愿服务常态化。深入实施全民科学素质行动计划，加强科普宣传教育，支持开展哲学社会科学研究。编纂出版《苏州市志》、《苏州通史》和《吴郡文编》，建成市档案馆新馆。增强公共文化服务能力，持续开展文化惠民活动，建成国家公共文化服务体系示范区，人均拥有公共文体设施面积超过2.8平方米。发扬吴文化优秀传统，保护传承物质和非物质文化遗产。修复古典园林，改善古典园林周边环境，保持世界遗产的完整性和真实性，做好大运河苏州古城段、江南水乡古镇申报世界文化遗产工作。繁荣文艺创作，办好太湖文化论坛、中国苏州文化创意设计产业交易博览会、中国长江文化艺术节等重大活动。重点发展创意设计、出版发行、新闻传媒等产业，建成中国光华（苏州）文化创意产业园、阳澄湖数字文化创意产业园等文化产业集聚区以及现代传媒广场、国际影视娱乐城等项目，提升文化产业发展水平，文化产业增加值占地区生产总值的比重超过7%。实施中长期教育改革和发展规划纲要，教育发展主要指标达到发达国家本世纪初平均水平。增加教育投入，优化投入结构。改革教育管理体制和办学机制，完善现代学校制度，倡导教育家办学，鼓励通过购买服务等方式支持民办教育发展。深化素质教育，加强教师队伍建设，加快教育国际化、信息化进程，培育教育特色品牌。推进基础教育优质均衡发展，新建、改建一批幼儿园和中小学校，形成与人口结构、人口分布相适应的学校布局，着力解决不断增长的教育需求与教育供给之间的矛盾。优化发展职业教育，不断完善适应产业结构调整要求、体现终身教育理念、普职互通、中高职衔接的现代职业教育体系。支持在苏高校发展，加强与国内外著名高校的合作办学，增强高等教育自主创新能力，更好地服务苏州现代化建设。继续做好市职业大学升本工作。倡导全民学习，发展社区教育、老年教育，构建学习型社会。

（七）突出改善民生质量，提升社会建设和管理水平

加强以改善民生为重点的社会建设，全力办好各项惠民实事。优化支出结构，城乡公共服务支出占财政支出的比重超过73%。深化收入分配制度改革，落实城乡居民收入六年倍增计划，切实提高新老苏州人的收入水平，努力实现居民收入增长与经济发展同步、劳动报酬增长与劳动生产率提高同步。实施更加积极的就业政策，开发就业岗位，加大就业援助力度，切实解决困难人群的就业问题。支持自主创业，建成国家级创业型城市。预防和化解劳动人事争议，保障劳动者合法权益。扩大社会保障覆盖面，逐步提高保障水平，确保城乡养老保险覆盖率、医疗保险覆盖率均超过99%，重病大病人员全部享有较高水平的医疗救助，城乡60周岁以上老年人全部纳入社会化服务。健全医疗救助、自然灾害救助和临时救助体系，建立适度普惠型社会福利制度。推进慈善公益、红十字事业发展。建成市社会福利总院。合理布局养老设施，提升养老服务质量，促进养老服务社会化，每千名老年人拥有机构养老床位数达到42张。拓展残疾人救助领域，提高残疾人社会保障和服务水平。落实住房保障制度，加强保障性住房建设和管理，扩大住房公积金制度覆盖面和受益面。完成南环新村解危改造、城区居民家庭“改厕”和第二轮老住宅小区综合整治任务，继续实施危旧房和城中村（无地队）改造。科学规划和建设城市停车场，新建一批公共绿地等城市开敞空间。坚持公交优先，改进公交服务，推行智能化管理，提高市民公交出行分担率。认真执行食品、药品准入制度，严格实行监督管理，保障群众饮食用药安全。稳定粮食生产，完成地方粮食储备任务。落实“菜篮子”市长负责制，加快城乡农贸市场升级改造，完善农产品平价直销体系。强化价格调控与监管，努力保持物价总水平基本稳定。加大卫生事业投入力度，健全社区卫生服务和卫生监督、疾病防控、妇幼保健、医疗急救等体系，建成市公共医疗中心和苏州大学附属第一医院迁建项目，每千人拥有医生数达到2.32人。加强慢性病综合防治，优化老年人医疗保健服务。发扬吴门医派传统，振兴中医药事业。深入开展爱国卫生运动和健康城市建设。增加公共体育设施，提升市民科学健身水平。发展竞技体育，办好第53届世界乒乓球锦标赛，力争苏州体育健儿在国内外重大赛事上获得优异成绩。继续实行人口与家庭发展公共服务政策和计划生育利益导向政策，推进幸福家庭世代服务工程，促进优生优育，提高人口素质。贯彻党的民族宗教政策，增进民族团结、宗教和谐。坚持男女平等、儿童优先，实施妇女儿童“十二五”发展规划，推动妇女儿童事业发展。扎实开展法治苏州建设，弘扬法治文化，营造良好的法治环境，深化法治惠民，扩大法律援助范围，争创首批江苏省法治城市和全国“六五”普法先进单位。加强和创新社会管理，增强基层民主自治能力，夯实基层基础，城乡和谐社区建设达标率分别超过98%和95%。构建社会信用系统，依法建立信用信息的采集、披露和共享机制。深化平安苏州建设，构筑更加严密的社会治安防控体系，严厉打击各类严重刑事犯罪活动和境内外敌对势力的渗透破坏活动。健全矛盾纠纷大调解体系，发挥人民调解的基础性作用，强化信访工作责任，及时疏导和化解人民内部矛盾。严格执行安全生产责任制，进一步落实企业安全生产主体责任，突出抓好重点行业、重点领域的安全监管，严防严控重特大事故和重大影响事故。完善应急管理体制，提高突发事件监测预警和应急处置能力。加强国防教育、国防动员和民兵预备役工作，支持驻苏解放军和武警部队建设，加大拥军办实事力度，争创全国双拥模范城“六连冠”。

（八）全面构建两型社会，高标准推进生态文明建设

牢固树立绿色低碳理念，持之以恒抓好环境保护和生态修复，建设资源节约型、环境友好型社会。切实加强水污染防治，完成东太湖综合整治、走马塘延伸拓浚、七浦塘拓浚等工程和太湖流域水污染治理阶段性任务，整治城乡河道，提高水功能区水质达标率。加大水源地保护力度，确保饮用水达标。推进污水处理系统建设和升级改造，力争城镇生活污水处理率达到95%，农村生活污水处理率达到70%，其中太湖、阳澄湖保护区达到85%。促进污水处理厂污泥无害化处理。综合治理大气污染，普遍实施PM2.5监测，努力减少灰霾天气。开展城市声环境功能区达标建设，控制噪声污染。加强核与辐射环境监测和安全监管，防治重金属污染，规范固体废物处置管理，防范环境风险。治理工业点源、农业面源污染，有效削减污染物排放。推行生活垃圾分类收集，促进无害化处理。严格执行环境评价准入制度，严禁新上高耗能、高排放项目。改造传统产业，淘汰落后产能。建设节能工程，推行合同能源管理，深化“企业节能低碳行动”和“能效之星”创建活动。推广节能建筑和绿色建筑，做好公共机构节能示范工作。认真实施土地利用总体规划，优化土地利用结构，提高土地利用效益，综合利用城市地下空间资源。建设循环经济重点工程，广泛推行清洁生产，建成再生资源回收利用体系，积极争取国家低碳城市试点。落实生态补偿措施，推进“绿色苏州”建设，实施三角咀湿地公园等重大生态保护和修复工程，加强森林、湿地、风景名胜区和自然保护区管理，确保我市率先进入国家生态市和国家生态园林城市行列，建成一批全国生态文明城市和生态文明园区，使苏州成为宜居宜业的幸福家园。

（九）切实抓好自身建设，努力打造人民满意的政府

加快转变职能，改进行政管理，提升工作效能和服务水平。深化行政审批制度改革，加强行政服务中心标准化建设，进一步削减审批事项，规范审批行为，优化审批流程，提高审批效率。推进经济发达镇行政管理体制改革，探索产业园区和所在镇“区镇合一”行政管理体制改革。严格依法行政，切实按照法定权限和法定程序行使权力、规范行为。高质量做好政府立法工作，全面开展规章和规范性文件实施情况评估。强化行政执法责任制，抓好相对集中行政许可权试点工作，规范行政裁量权。贯彻执行行政执法案卷评查和重大行政处罚、重大行政许可备案审查制度。依法开展行政复议和应诉，推行行政指导、行政调解。深化作风效能建设，力倡主动服务、创新服务、尽责服务、高效服务、廉洁服务，实施项目化推进，积极创建服务品牌。深入开展纳税人评议政风行风活动，加强对领导干部、机关部门以及直接服务企业、服务群众的重点处室的作风考评，进一步优化经济社会发展环境。运用信息化手段，整合热线资源，集聚服务事项，把便民服务中心建设成为集政策咨询、信息查询、服务指南、业务办理、投诉建议、监督评议为一体的阳光服务平台。注重教育培训，加强绩效考核，完善激励机制，建设业务过硬、作风正派的公务员队伍。规范各类论坛、庆典、研讨会等活动，精简会议、文件。严控行政经费支出，确保因公出国（境）经费、公务用车购置及运行费、公务接待费支出零增长。认真落实党风廉政建设责任制，完善同苏州率先基本实现现代化相适应的惩治与预防腐败体系，着力查办违法违纪案件。针对工程建设、政府采购、招标投标、房地产开发、土地使用权出让等领域的突出问题，进行专项治理，规范运作流程，维护社会主义市场经济秩序，推动反腐倡廉建设。深化政务公开，促进行政权力公开透明运行，保障群众的知情权、参与权、表达权、监督权。抓好对财政性资金、重大政策措施、民生实事工程的审计和经济责任审计，推进审计整改和审计结果公开。切实执行人民代表大会及其常务委员会的决定和决议，主动接受人大的法律监督、工作监督和政协的民主监督，接受司法机关的监督，接受新闻舆论的监督，接受以网络监督为重点的社会监督。认真实施人大议案，提高代表建议和政协提案的办理质量。

各位代表，当前的经济形势相当严峻，政府的工作任务相当艰巨。为了全面实现今年的各项目标，新一届政府要充分认识保持经济平稳较快发展是率先基本实现现代化的基本前提和重要内容，牢牢把握稳中求进的总基调，把更大气力、更多精力放在稳增长上，一着不让抓落实，千方百计扩成效，全力以赴促发展。把发展实体经济作为稳增长最关键的环节，夯实经济增长的基础；把稳住外贸作为稳增长最紧迫的任务，推动开放型经济持续发展；把扩大有效投入作为稳增长最有力的措施，尽快形成新的增长点；把促进消费作为稳增长最持久的动力，更好地发挥消费对经济的拉动作用；把帮扶企业作为稳增长最直接的途径，确保政策落实到位、指导服务到位、环境营造到位。在保持和提升苏州经济领先地位的同时，围绕快转型，将转变经济发展方式作为重中之重，优化产业结构，推动科技创新，狠抓节能减排，提高经济运行质量；围绕添活力，开展重点领域和关键环节的改革攻坚，力争取得新的突破，构建未来发展的新优势；围绕促和谐，深入细致地抓好社会稳定、安全生产、应急管理等工作，维护安定祥和的良好局面；围绕惠民生，大力实施富民工程，加快发展社会事业，努力让广大群众早得益、多受惠。今年是苏州率先基本实现现代化的突破之年，新一届政府务必保持昂扬向上的精神风貌、奋发有为的进取意识、敢打敢拼的工作劲头、志在必得的坚定信念，与全市人民一起，同心协力、埋头苦干，确保“第二个率先”首战必胜、首战告捷。

各位代表，时代赋予重任，人民寄予厚望。让我们紧密团结在以胡锦涛同志为总书记的党中央周围，高举中国特色社会主义伟大旗帜，全面贯彻落实科学发展观，在中共苏州市委的坚强领导下，扎实工作，开拓创新，为加快“三区三城”建设，率先基本实现现代化，早日建成宜居新苏州、创业新天堂、幸福新家园而努力奋斗！
